# A Variant Origin of the Carotid Sinus Nerve

**DOI:** 10.7759/cureus.2883

**Published:** 2018-06-26

**Authors:** Kevlian Andrew, Joe Iwanaga, Marios Loukas, Rod J Oskouian, R. Shane Tubbs

**Affiliations:** 1 Anatomical Sciences, St. George's University, St. George, GRD; 2 Seattle Science Foundation, Seattle, USA; 3 Anatomical Sciences, St. George's University, St Georges, GRD; 4 Neurosurgery, Swedish Neuroscience Institute, Seattle, USA; 5 Neurosurgery, Seattle Science Foundation, Seattle, USA

**Keywords:** glossopharyngeal nerve, cranial nerve ix, intercarotid plexus, vagus nerve

## Abstract

The carotid sinus nerve is known to convey baroreceptive fibers from the carotid sinus. Despite studies on the baroreflex pathway and the course and communications of the carotid sinus nerve with the surrounding nervous and vascular structures, there have been scant reports on variations in the origin of the carotid sinus nerve (CSN). We identified an unusual origin of the CSN. On the right side of a cadaveric specimen, the CSN was found to arise from two small rami extending from the external laryngeal nerve. Such a case can help better understand various pathways used to monitor the carotid sinus. Additionally, surgeries that manipulate the superior laryngeal nerve could possibly injure a variant carotid sinus nerve, as seen in the present case.

## Introduction

The carotid body is often found on the posterior wall of the carotid artery at the level of its bifurcation, usually at C4 [1]. The carotid sinus is a small dilatation [2] in the common carotid artery, most commonly at the origin of the internal carotid, but infrequently at the end of the common carotid or the beginning of the external carotid artery [2-3]. The carotid body and sinus are a chemoreceptor and a baroreceptor, respectively [1]. The carotid sinus nerve (CSN), a contribution to the so-called intercarotid plexus (not to be confused with the internal carotid plexus, which is a sympathetic-only plexus), is the primary innervation of the carotid sinus. The carotid body and sinus are innervated by the intercarotid plexus, which is found within the carotid triangle [2,4]. The intercarotid plexus is made up of contributions from cranial nerves (CN IX and CN X) and the cervical sympathetic trunk—with rare hypoglossal nerve contribution. Medially, the intercarotid plexus can be connected to the pharyngeal plexus [2]. The CSN typically originates from the glossopharyngeal nerve, which relays baroreceptive information to the nucleus tractus solitarius [1,3]. Herein, we describe an unusual origin of the CSN and review the literature regarding other possible sources of this branch to the carotid sinus.

## Case presentation

**Figure 1:**
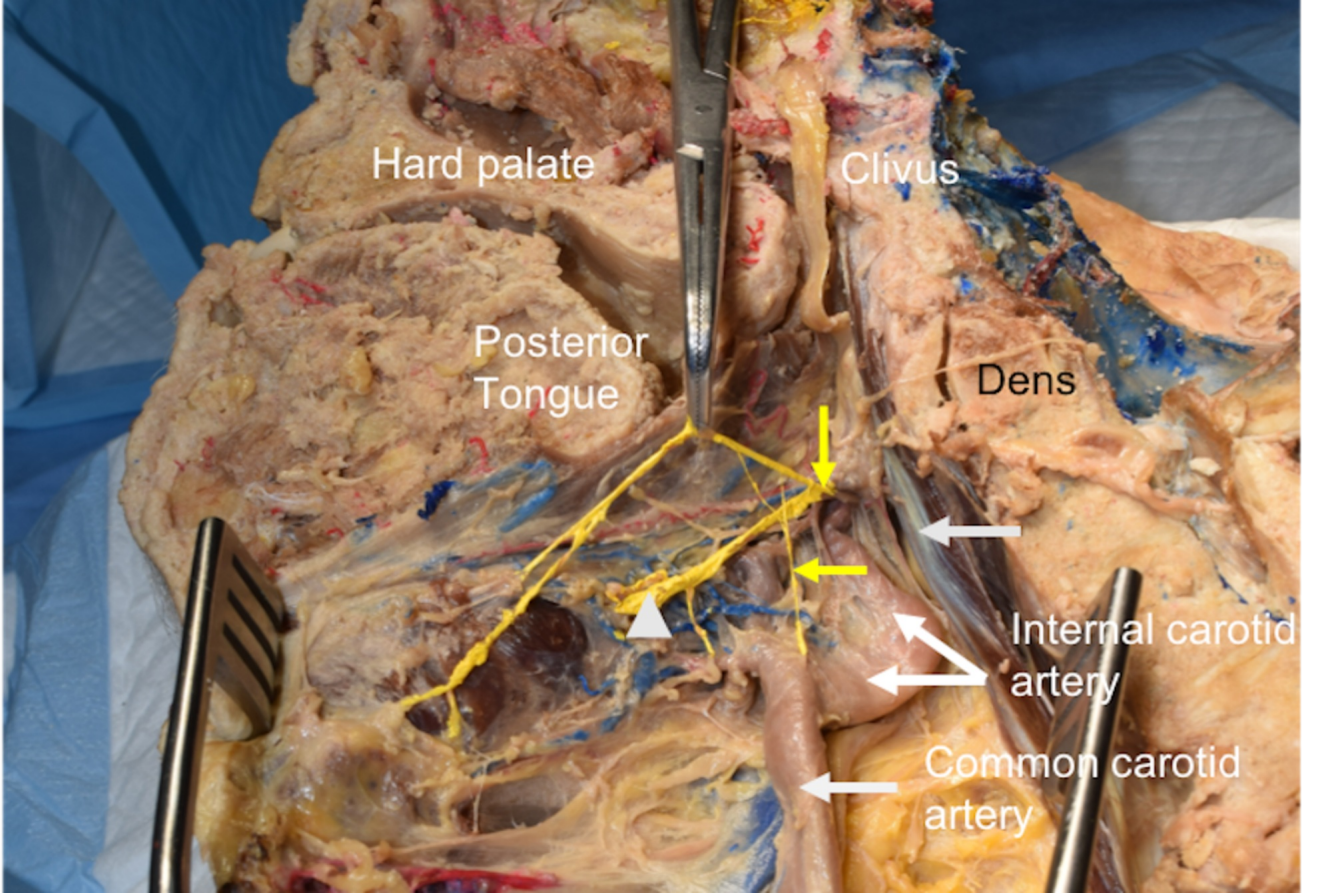
Medial view of the specimen reported here. Note the superior laryngeal nerve (vertical yellow arrow), internal laryngeal nerve (elevated with hemostats), and internal laryngeal nerve (white arrowhead). Observe that the CSN (horizontal yellow arrow) is arising from the external laryngeal nerve to then descend onto the carotid sinus noted here as a swelling at the origin of the internal carotid artery. CSN: carotid sinus nerve

**Figure 2:**
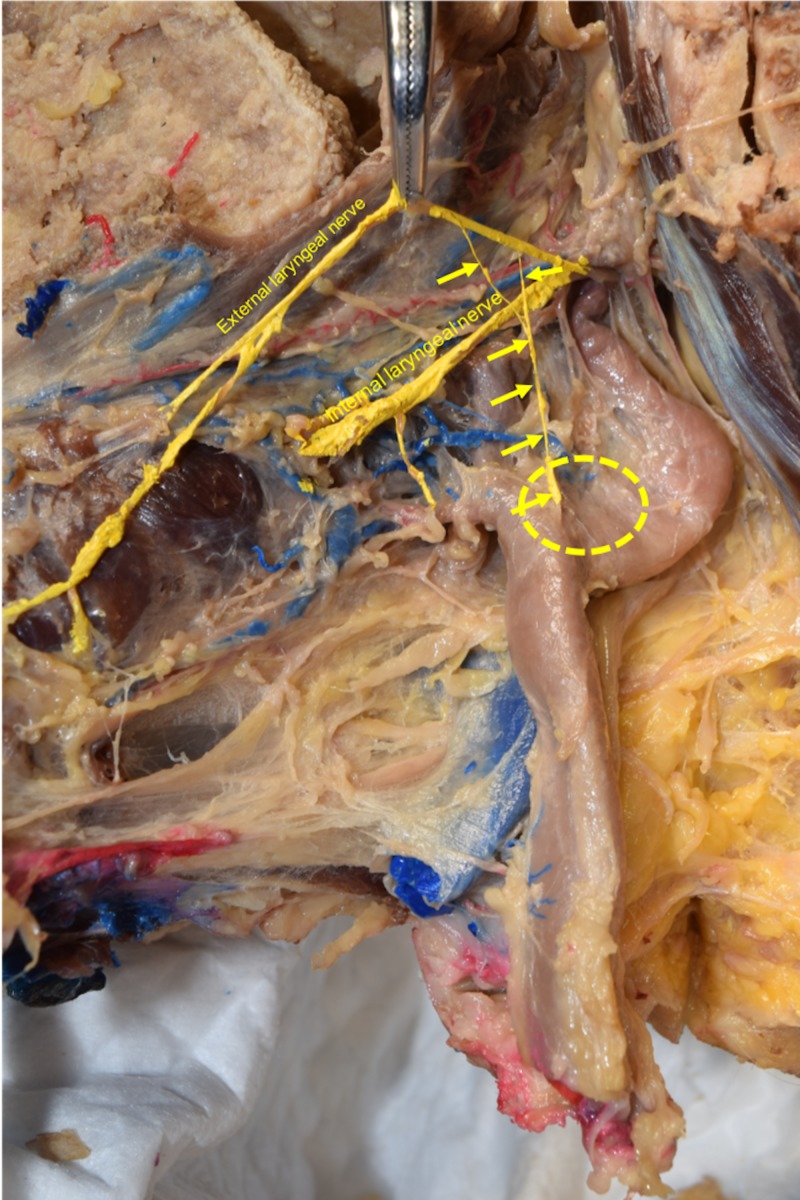
Zoomed-in view of Figure 1. Note the two rami (upper two arrows) contributing to the formation of the CSN (lower four arrows). The region of the carotid sinus is outlined by the yellow dotted circle. CSN: carotid sinus nerve

During the routine dissection of the neck in an embalmed Caucasian female cadaver, an unusual origin of the CSN was identified. This specimen was 82-years-old at death and had undergone latex injection of the vessels of the head and neck via cannulation of the common carotid artery and internal jugular vein. The specimen was hemisected in the sagittal plane. From a medial to lateral dissection, the common carotid artery was identified and specifically the carotid sinus. Next, regional nerves were dissected, including the superior laryngeal nerve and its branches, the internal and external laryngeal nerves. On the right side, the external laryngeal branch of the superior laryngeal nerve was found to give rise to the CSN via two small rami (Figures 1-2). The external laryngeal nerve then continued in a normal fashion, medial to the superior thyroid artery, and innervated the cricothyroid and inferior pharyngeal constrictor muscles. No other anatomical variations were noted in this region.

## Discussion

Our case is an example of a very rare origin of the CSN from the external laryngeal nerve. The CSN—known also as the ramus descensus glossopharyngei of Braeucker, intercarotid nerve of De Castro, Hering’s nerve, carotid sinus nerve proper, and ramus caroticus glosspharyngei [1,4-6]—is one of the main components of the intercarotid plexus as mentioned earlier. The external laryngeal nerve, a branch of the superior laryngeal nerve, is a significant branch of the vagus within the neck, as it innervates the cricothyroid and inferior pharyngeal constrictor muscles [1]. Interestingly, the deep trunk of the intercarotid plexus is formed when a branch of the superior cervical sympathetic ganglion receives a communicating branch from the superior laryngeal nerve.

Variant origins of the CSN

According to Tubbs et al. [1], 48 out of 58 specimens (82.8%) were found to have the CSN arising from the glossopharyngeal nerve or one of its branches (pharyngeal branch or branch to the stylopharyngeus). In the study by Sheehan et al. [2], all 33 specimens had the CSN branching directly from the glossopharyngeal nerve. However, these authors did note differences in the exact point of branching from the glossopharyngeal nerve. According to the study by Toorop et al. [6], out of 12 cadaveric specimens, all had a distinct CSN arising from the glossopharyngeal nerve. Fuse [4] found that 31.3% of CSNs descend as two trunks. Tubbs et al. [1] found a duplicated CSN in 33% of individuals, Sheehan et al. [2] noted this in two of 33 specimens, and Toorop et al. [6] identified this in two of their specimens but observed these two nerves later joining to form one CSN and this was a similar finding to the study by Sheehan et al. [2]. Previous studies have suggested that the CSN may be one of the pharyngeal branches of CN IX [2]. One study mentioned that a small branch of the glossopharyngeal nerve can join a branch of the vagus nerve to form the CSN [7].

The CSN has also been found to have distinct communications along its descent [1] with branches of surrounding nerves. These include the vagus nerve and its branches; the pharyngeal branch (most commonly reported communication) [4] and the superior laryngeal nerve branch; the cervical sympathetics, and the hypoglossal nerves (rarest communication) [1,4,6,8-10]. The carotid sinus contribution from the vagus nerve is thought to occur via the intercarotid plexus. Other branches from the vagus nerve that might contribute to the intercarotid plexus include branches from its inferior ganglion and pharyngeal branch.

## Conclusions

Such a case as presented herein can help better understand various pathways used by the CSN. Additionally, surgeries that manipulate the superior laryngeal nerve could possibly injure a variant CSN as seen in the present case.
